# Effects of Sublethal Concentrations of Pyridaben on Development, Reproduction, and *Vg* Gene Expression in *Neoseiulus womersleyi*

**DOI:** 10.3390/insects17010116

**Published:** 2026-01-20

**Authors:** Juan Wei, Chengcheng Li, Cancan Song, Xinyue Yang, Chunxian Jiang, Qing Li

**Affiliations:** College of Agronomy, Sichuan Agricultural University, Chengdu 611130, China; juanwei666@163.com (J.W.); cc0394@outlook.com (C.L.); songcan1993@163.com (C.S.); cazsyy0825@163.com (X.Y.)

**Keywords:** *Neoseiulus womersleyi*, pyridaben, sublethal effects, *Vg* gene, RNAi

## Abstract

The predatory mite *Neoseiulus womersleyi* is a key natural enemy in Integrated Pest Management (IPM), but its efficacy is threatened by non-target effects of acaricides like pyridaben. This study evaluated the transgenerational sublethal effects of pyridaben (LC_30_ and LC_50_) on *N. womersleyi* and explored the role of vitellogenin (*Vg*) genes. Using two-sex life table analysis, we found that exposure reduced longevity, fecundity, and oviposition period in F_0_ females, while prolonging immature development and suppressing population growth parameters (*r*, *λ*) in F_1_. Two *Vg* genes (*NwVg1* and *NwVg2*) were cloned and characterized; their expression was significantly downregulated by pyridaben. RNAi-mediated silencing of *NwVg1* or *NwVg2* recapitulated the pyridaben-induced reproductive impairments, confirming their functional role. Our results demonstrate that pyridaben imposes multigenerational costs and that its reproductive toxicity is mediated, at least in part, through the suppression of Vg synthesis.

## Simple Summary

Predatory mites are important natural enemies used in agriculture to control pest mites. However, they can be harmed by chemical pesticides, even at low concentrations that are not immediately lethal. This study investigated how low (sublethal) concentrations of a common acaricide, pyridaben, affect a beneficial predatory mite, *Neoseiulus womersleyi*. We found that exposure to these sublethal doses reduced the lifespan and egg-laying ability of the directly exposed generation (F_0_). It also negatively impacted the next generation (F_1_), slowing down their development, reducing survival of young mites, and ultimately suppressing the population’s growth rate. For the first time, we identified and studied two key genes in this mite, *NwVg1* and *NwVg2*, which are crucial for egg production. Pyridaben exposure lowered the activity of these genes. When we experimentally turned off these genes, the mites showed similar problems in reproduction and survival as those exposed to the pesticide. Our results show that pyridaben can seriously harm this beneficial predator by damaging its health and disrupting its reproductive genes.

## 1. Introduction

The predatory mite *Neoseiulus womersleyi* was first discovered by Schicha on strawberries in Australia in 1975; this species is now widely distributed across numerous regions including China, Korea, Japan, the Philippines, Australia, New Zealand, and Russia [1,2,3,4,5,6,7,8]. Valued for its high reproductive capacity and effective predation, it has become a cornerstone of integrated pest management (IPM) strategies. However, the field efficacy and population sustainability of such beneficial arthropods are fundamentally threatened by the non-target effects of broad-spectrum chemical pesticides [9].

Among these pesticides, the acaricide pyridaben represents a prevalent and significant concern. As a mitochondrial complex I inhibitor, it is highly effective against pest mites and is consequently one of the most extensively used acaricides globally and in China [10]. Although pyridaben’s acute toxicity to various natural enemies has led to its general classification as incompatible with IPM [11], this broad label may obscure subtler yet critical risks. A comprehensive ecological risk assessment must go beyond acute lethality to elucidate its sublethal effects, which can chronically impair predator physiology and population growth without causing immediate death [12]. For many important species, including *N. womersleyi*, the nature and mechanisms of these sublethal impacts, particularly those targeting reproductive physiology, remain inadequately characterized.

Sublethal pesticide exposure can compromise arthropod fitness through multifaceted impairments in development, reproduction, and behavior [13]. For predatory mites, research confirms that pyridaben can reduce fecundity, alter developmental timing, and diminish predatory capacity [14,15]. Despite these well-documented phenotypic consequences, a critical knowledge gap persists. Most studies remain at the level of describing organismal or population-level outcomes. The underlying molecular mechanisms that initiate these cascades of reproductive impairment in predatory mites remain largely unexplored. Bridging this gap between toxicant exposure, molecular initiation, and phenotypic outcome is essential for a mechanistic understanding of pesticide toxicity.

We hypothesize that the reproductive disruption caused by pyridaben may be mediated through the suppression of vitellogenin (Vg) synthesis. Vg, the principal yolk protein precursor, is fundamental to oocyte maturation and embryonic nutrition in oviparous arthropods, making its regulated synthesis a critical node for reproductive success [16,17]. This hypothesis is grounded in two convergent lines of evidence. First, the endocrine regulation of Vg in mites exhibits distinct characteristics that may heighten its vulnerability. Unlike most insects where *Vg* is primarily regulated by juvenile hormone, evidence suggests that in mites, *Vg* synthesis is closely linked to ecdysteroid signaling pathways [18,19,20], a system that could be particularly susceptible to chemical interference. Second, and more directly, there is precedent that acaricides can target this pathway; for instance, scoparone has been shown to suppress *Vg* gene expression and impair reproduction in pest mites [21,22,23]. Therefore, the present study was designed to test the hypothesis that pyridaben exerts its sublethal reproductive toxicity on the predatory mite *N. womersleyi* through the disruption of Vg synthesis, thereby bridging the mechanistic gap between chemical exposure and phenotypic impairment.

To this end, we conducted a multi-level investigation into the transgenerational effects of sublethal pyridaben concentrations on *N. womersleyi*. Our specific objectives are threefold: (1) to quantify the impact on demographic parameters across generations (F_0_ and F_1_) using age-stage, two-sex life table analysis; (2) to determine if sublethal exposure suppresses the expression of key *Vg* genes (*NwVg1* and *NwVg2*); and (3) to directly validate the functional role of these *Vg* genes in reproduction via RNA interference (RNAi). This integrated approach, spanning population demography, molecular expression, and functional genetics, aims to deconstruct the chain of events from chemical exposure to population consequence. The findings will provide a comprehensive mechanistic understanding of pyridaben’s sublethal toxicity, offering novel insights for the refined risk assessment of acaricides and the conservation of biocontrol agents within sustainable agriculture frameworks.

## 2. Materials and Methods

### 2.1. Insect Source and Reagents

The predatory mite *N. womersleyi* was collected in 2021 from soybean leaves at Sichuan Agricultural University. A colony of the prey mite *T. urticae* was maintained on soybean plants (var. Zhenong 8). Both species were reared in an intelligent climate chamber (25 ± 1 °C, 75 ± 5% RH, 16L:8D photoperiod) using a water-sealed dish method without pesticide exposure. Briefly, sterilized glass dishes were lined with a layer of sterile absorbent cotton (approx. 5 mm thick), moistened with distilled water. Clean soybean leaf disks were placed on the cotton and inoculated with mites. Cotton, leaves, and prey were refreshed regularly to maintain hygiene and prevent contamination. Prior to experiments, the identity of the *N. womersleyi* colony was confirmed via phylogenetic analysis of ITS and COI sequences against reference strains. Technical-grade pyridaben (97% purity; Xinyi Taisong Chemical Co., Ltd., Xuzhou, China) was dissolved in acetone and diluted with distilled water containing 0.05% Tween-80 (*v*/*v*) to prepare working solutions. A Potter precision spray tower (Burkard Manufacturing Co. Ltd., Rickmansworth, UK) was used for topical application to ensure uniform coverage.

### 2.2. Effects of Sublethal Concentrations of Pyridaben on the Development and Reproduction of F_0_ and F_1_ Generations of N. womersleyi

Based on a prior bioassay [24], the LC_30_ and LC_50_ for adult female *N. womersleyi* were 24.417 and 37.395 μg/mL, respectively. These sublethal concentrations were used for all subsequent experiments.

F_0_ generation exposure: Newly emerged, age-synchronized adult females were topically treated with either LC_30_, LC_50_, or a solvent-only control (a mixture of distilled water and acetone containing 0.05% Tween-80, *v*/*v*) (*n* = 60 per group). After 24 h, survivors were individually transferred to six-well plates containing a *T. urticae*-infested leaf disk on a moistened filter paper/cotton base, each paired with an untreated male. Female longevity and daily egg production were recorded until death.

F_1_ generation assessment: Eggs laid by F_0_ females on the same day were collected and reared individually using the same protocol as for the F_0_ generation (i.e., in six-well plates with prey provision), but without any pyridaben exposure. Upon maturation, F_1_ individuals were paired (one female + one male) to construct an age-stage, two-sex life table (*n* = 60 pairs per original F_0_ treatment group, including the solvent-only control). The developmental duration of each stage, adult longevity, and fecundity were recorded until death for both sexes.

### 2.3. RNA Extraction, Gene Cloning, and Expression Analysis

Sample collection: For gene expression analysis, age-synchronized adult females were exposed to LC_30_, LC_50_, or control as in Section 2.2. After 24 h, 50 females per treatment were pooled as one biological replicate (3 replicates per treatment), flash-frozen, and stored at −80 °C.

RNA extraction and cDNA synthesis: Total RNA was extracted using a FastPure Cell/Tissue Total RNA Isolation Kit (Vazyme, Nanjing, China). cDNA was synthesized using the HiScript III First Strand cDNA Synthesis Kit (Vazyme).

*Vg* gene cloning and sequence analysis: To obtain full-length sequences for primer design and functional annotation, *NwVg1* and *NwVg2* were amplified from cDNA using gene-specific primers designed based on NCBI entries (KX620366.1, KX620367.1) (Table S1). PCR products were cloned using a TA/Blunt-Zero Cloning Kit (Vazyme) and sequenced. Bioinformatic analysis (domain prediction, phylogeny) was performed using standard tools (ProtParam https://web.expasy.org/protparam/, accessed on 16 October 2024), the Conserved Domain Database (CDD, https://www.ncbi.nlm.nih.gov/cdd/, accessed on 16 October 2024), and MEGA11 (v11.0.13).

Quantitative PCR (qPCR): Gene-specific qPCR primers were designed (Table S2). Reactions were performed using ChamQ SYBR qPCR Master Mix (Vazyme) on a QuantStudio system. The β-actin gene was the reference. Relative expression was calculated via the 2^−ΔΔCt^ method. Three biological replicates, each with three technical replicates, were analyzed per treatment.

### 2.4. RNA Interference (RNAi) Functional Assay

dsRNA Synthesis and Purification: Gene-specific fragments (~500 bp) of *NwVg1*, *NwVg2*, and *GFP* (control) were amplified using T7 promoter-linked primers (Table S3). The PCR products were purified and used as templates for in vitro transcription with the T7 RNAi Transcription Kit (Vazyme), following the manufacturer’s instructions. Following synthesis, the dsRNA reactions were treated with DNase I and RNase T1 to remove template DNA and single-stranded RNA, respectively. The dsRNA was then purified via ethanol precipitation, resuspended in nuclease-free water, and its concentration and purity were verified using a NanoDrop 2000 spectrophotometer (Thermo Fisher Scientific, Waltham, MA, USA). The final working concentration was adjusted to 1000 ng/µL.

dsRNA Feeding and Reproductive Bioassay: Age-synchronized adult females were starved for 24 h and then fed droplets of a 25% sucrose solution containing 1000 ng/μL dsRNA, following Ghazy et al. [25]. Feeding was confirmed by observing the ingestion of a carmine red dye mixed into the solution. For each dsRNA type, 30 successfully fed females were individually paired with a male on a prey-infested leaf disk (*n* = 30). The interval from feeding to first oviposition, longevity, and total fecundity were recorded.

Verification of Gene Silencing Efficiency: To confirm RNAi efficacy, a separate cohort of females was fed dsRNA as described above. Twenty-four hours after the completion of feeding, a total of 150 successfully fed females per treatment group (pooled into three biological replicates of 50 individuals each) were collected. Total RNA was extracted, and cDNA was synthesized. The relative expression levels of *NwVg1* and *NwVg2* were quantified by qPCR (as per Section 2.3), using the dsGFP-treated group as the calibrator. A significant reduction in target gene mRNA levels confirmed successful gene silencing.

### 2.5. Data Analysis

Life Table and Statistical Analysis: Data on the life-history parameters of the F_0_ generation (female adult longevity, pre-oviposition period, oviposition period, and fecundity) were tested for normality (Shapiro–Wilk test) and homogeneity of variances (Levene’s test). As these assumptions were met, the data were analyzed using one-way analysis of variance (ANOVA). In cases where the ANOVA indicated a significant effect (*p* < 0.05), Tukey’s Honestly Significant Difference (HSD) test was applied for pairwise comparisons (SPSS 27.0). For the F_1_ generation, population parameters-including the net reproductive rate (*R*_0_), intrinsic rate of increase (*r*), finite rate of increase (*λ*), and mean generation time (*T*)—were calculated and compared using the age-stage, two-sex life table analysis in the TWOSEX-MSChart program [26,27]. Means and standard errors were estimated via the bootstrap method with 100,000 resamplings. Significant differences (*p* < 0.05) among treatments for these population parameters were determined using the paired bootstrap test within the same software, which is a non-parametric method not reliant on the same distributional assumptions as ANOVA.

Gene Expression Analysis: The relative expression levels of *NwVg1* and *NwVg2*, calculated via the 2^−ΔΔCt^ method from three biological replicates, were also checked for normality and homoscedasticity. Subsequently, the data were analyzed by one-way ANOVA followed by Tukey’s HSD test for multiple comparisons (GraphPad Prism 9.5.1).

Bioinformatic Analysis: For the cloned *NwVg1* and *NwVg2* sequences, standard bioinformatic analyses were performed for protein prediction, conserved domain identification, and similarity searches using BLAST (version 2.15.0). A phylogenetic tree was constructed using the Neighbor-Joining method in MEGA 11.0 with 1000 bootstrap replicates. Detailed resources and sequence information are provided in Tables S4 and S5.

## 3. Results

### 3.1. Effects of Sublethal Concentrations of Pyridaben on Life Table Parameters of F_0_ Female Adults of N. womersleyi

The effects of pyridaben at LC_30_ and LC_50_ on the life table parameters of F_0_ female adults of *N. womersleyi* are summarized in Table 1. The results indicate that exposure to sublethal concentrations of pyridaben led to significant alterations in the longevity, pre-oviposition period, oviposition period, and fecundity of the F_0_ female adults compared to the control group. One-way ANOVA revealed that the treatment had a significant effect on female longevity (*F* = 384.52, *p* < 0.001), pre-oviposition period (*F* = 183.41, *p* < 0.001), oviposition period (*F* = 295.67, *p* < 0.001), and fecundity per female (*F* = 644.33, *p* < 0.001). Specifically, longevity, total fecundity, and the oviposition period were significantly reduced, while the pre-oviposition period was significantly prolonged in a concentration-dependent manner.

A clear dose-dependent effect was observed between the two sublethal concentrations. As the concentration of pyridaben increased, the negative impacts on life parameters became more pronounced. Compared to the control, both LC_30_ and LC_50_ treatments significantly decreased the longevity, oviposition period, and fecundity per female, and significantly extended the pre-oviposition period.

In detail, the LC_30_ and LC_50_ treatments resulted in reductions in female adult longevity by 19.24% and 28.37%, respectively. The oviposition period was significantly shortened by 19.59% and 26.25%, and the mean fecundity per female was significantly reduced by 16.37% and 56.67%, respectively.

### 3.2. Effects of Sublethal Concentrations of Pyridaben on the Life Table Parameters of the F_1_ Generation of N. womersleyi

#### 3.2.1. Effects on the Developmental Duration of F_1_ *N. womersleyi*

The effects of LC_30_ and LC_50_ of pyridaben on the developmental duration of the F_1_ generation of *N. womersleyi* are presented in Table 2.

The results demonstrated that treatment with sublethal concentrations of pyridaben significantly prolonged the total developmental duration of F_1_ female mites in a concentration-dependent manner compared to the control (*F* = 24.67, *p* < 0.001). Furthermore, the longevity of female adults was significantly extended under the LC_30_ treatment (*F* = 11.23, *p* < 0.001). The prolongation of the total developmental duration in females was primarily attributable to the extended immature stages (*F* = 56.89, *p* < 0.001). Specifically, the egg stage (*F* = 45.62, *p* < 0.001) was significantly longer in both sublethal treatments, and the protonymph stage was also significantly extended under the LC_50_ treatment (*F* = 3.21, *p* = 0.045), relative to the control. However, the deutonymph stage did not show a significant difference among treatments (*F* = 0.00, *p* = 1.000).

In contrast, the total developmental duration of F_1_ males was not prolonged by sublethal pyridaben exposure. Instead, the longevity of male adults was significantly reduced compared to the control (*F* = 18.92, *p* < 0.001). However, the immature period of F_1_ males was significantly extended by the sublethal treatments (*F* = 17.89, *p* < 0.001). The egg (*F* = 35.41, *p* < 0.001), larva (*F* = 4.89, *p* = 0.010), and protonymph stages (*F* = 10.56, *p* < 0.001) of males were all significantly prolonged under both LC_30_ and LC_50_ treatments, whereas no significant difference was observed in the deutonymph stage compared to the control (*F* = 2.05, *p* = 0.135).

#### 3.2.2. Effects of Sublethal Concentrations of Pyridaben on the Reproduction of F_1_ Generation of *N. womersleyi*

The effects of LC_30_ and LC_50_ of pyridaben on the reproductive parameters of the F_1_ generation of *N. womersleyi* are summarized in Table 3. One-way ANOVA revealed that the pre-oviposition period (*F* = 13.12, *p* < 0.001) and oviposition period (*F* = 87.67, *p* < 0.001) of the F_1_ generation were significantly different among treatments; however, post hoc comparisons indicated that these parameters were not significantly affected by sublethal concentrations of pyridaben compared with the control. In contrast, the total pre-oviposition period was significantly prolonged in a dose-dependent manner (*F* = 2201.5, *p* < 0.001). Furthermore, the fecundity (number of eggs per female) was significantly different among treatments (*F* = 210.5, *p* < 0.001), with the LC_50_ treatment group being significantly lower than both the control and the LC_30_ treatment groups.

#### 3.2.3. Effects of Sublethal Concentrations of Pyridaben on Population Parameters in the F_1_ Generation of *N. womersleyi*

The sublethal concentrations of pyridaben ultimately significantly inhibited the population growth potential of the F_1_ generation (Table 4). Compared to the control, both LC_30_ and LC_50_ treatments significantly reduced the intrinsic rate of increase (*r*) and the finite rate of increase (*λ*), while significantly prolonging the mean generation time (*T*) and the population doubling time (*D_t_*). No significant differences in these parameters were observed between the two treatment concentrations. The net reproductive rate (*R*_0_) did not differ significantly among all groups.

This population-level inhibitory effect resulted from the integrated impact of the treatments on multiple aspects of individual survival and reproduction. Although the final proportion of individuals that successfully developed to the adult stage did not differ significantly among treatments (χ^2^ = 1.034, df = 2, *p* = 0.596), the age-stage, two-sex life table analysis revealed that sublethal treatments altered the dynamics of population survival and reproduction. This was evidenced by numerical reductions in stage-specific survival rates during the immature stages and changes in the peak timing and distribution of the population reproductive curves (Figure S1). These alterations collectively contributed to the decline in the aforementioned population growth parameters (*r*, *λ*).

### 3.3. Cloning of Vg Genes

In this experiment, two vitellogenin genes, *NwVg1* and *NwVg2*, were successfully cloned using cDNA of *N. womersleyi* as the template. PCR amplification followed by electrophoresis yielded single, distinct bands for each gene. Electrophoretic analysis showed that the fragment size of *NwVg1* was above 5000 bp, that of *NwVg2* was approximately 5000 bp, as compared to the DL5000 DNA marker. These results are consistent with the sequencing data provided by Sangon Biotech. The electrophoretic results are presented in Figure S2.

### 3.4. Bioinformatic Analysis of NwVg Genes

#### 3.4.1. Analysis of Full-Length Sequences and Encoded Amino Acid Characteristics

In this study, two vitellogenin genes, *NwVg1* and *NwVg2*, were successfully cloned from *N. womersleyi* for the first time. The gene sequences have been deposited in the NCBI database (Accession numbers: OR897817.1 for *NwVg1*, OR897819.1 for *NwVg2*). BLAST analysis revealed that *NwVg1* and *NwVg2* share the highest sequence identity with *NbVg1* and *NbVg2* from *N. barkeri*, reaching 92.18% and 93.38%, respectively. The characteristics of the full-length sequences and the encoded amino acids of the three obtained genes are summarized in Table 5.

#### 3.4.2. Prediction of Conserved Domains

The amino acid sequences of *NwVg1* and *NwVg2* were analyzed using the Conserved Domain Database (CDD) at NCBI. Both sequences were found to contain conserved domains characteristic of vitellogenin genes, including the DUF1943 (Domain of Unknown Function) superfamily and the VWD (von Willebrand factor type D) superfamily. Additionally, an extra PHA03247 superfamily domain was identified in *NwVg2*, spanning amino acids 367-1566, which encompasses the VWD domain located at amino acids 485–1003.

The DUF1943 superfamily, composed of several large open β-sheet structures, is a conserved domain present in Vg genes across many species, although its precise function remains uncharacterized. The VWD domain contains multiple type-D subdomains; among these, D1 and D2 are typically situated in the N-terminal propeptide, while other D subdomains contribute significantly to the formation of multimeric structures-a hallmark of *Vg* genes. The PHA03247 superfamily, often associated with the assembly or trafficking of large protein complexes, may reflect a distinct functional or structural feature specific to the *NwVg2* isoform.

**Figure 1:**
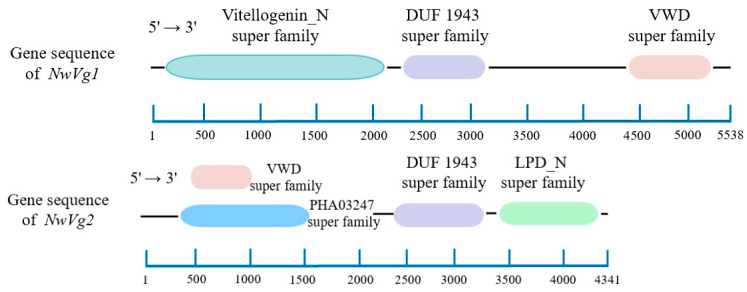
The conserved domains of genes *NwVg1* and *NwVg2*.

The identification of these characteristic conserved domains supports the reliability of the gene cloning performed in this study (Figure 1).

#### 3.4.3. Phylogenetic and Homology Analysis

**Figure 2:**
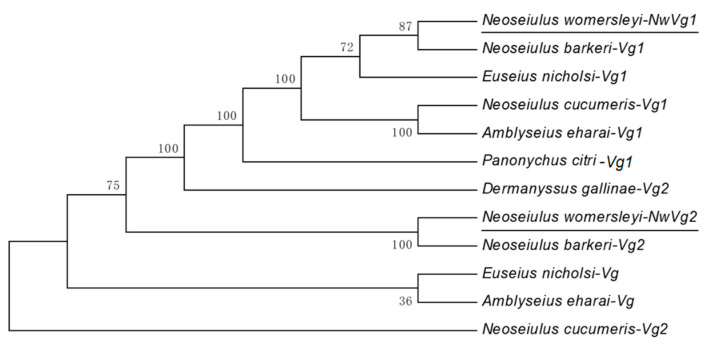
Phylogenetic tree of genes *NwVg1* and *NwVg2*.

A phylogenetic tree was constructed using the sequences of the three genes cloned in this study, along with vitellogenin gene sequences from other species retrieved from the NCBI database. The selected sequences included those from species with high similarity to *N. womersleyi* as well as evolutionarily distant species. Homology analysis was subsequently performed for *NwVg1* and *NwVg2*. The results are shown in Figure 2.

Phylogenetic analysis revealed that all *Vg1* genes clustered together in one distinct branch, while all *Vg2* genes formed a separate branch. Specifically, the *NwVg1* and *NwVg2* genes clustered with the corresponding *Vg1* and *Vg2* genes from *N*. *barkeri* with high bootstrap support (up to 100%), suggesting a common evolutionary origin. The results of the homology analysis indicate that the phylogenetic relationships of these *Vg* genes are consistent with their established taxonomic classification.

### 3.5. Relative mRNA Expression Levels

The results indicated that the mRNA expression of the *NwVg1* gene in *N. womersleyi* was significantly suppressed (*p* < 0.05) under both LC_30_ and LC_50_ treatments of pyridaben. The relative expression levels decreased by 41.29% and 51.02%, respectively. However, no significant difference (*p* > 0.05) in expression was observed between the two sublethal concentrations.

**Figure 3:**
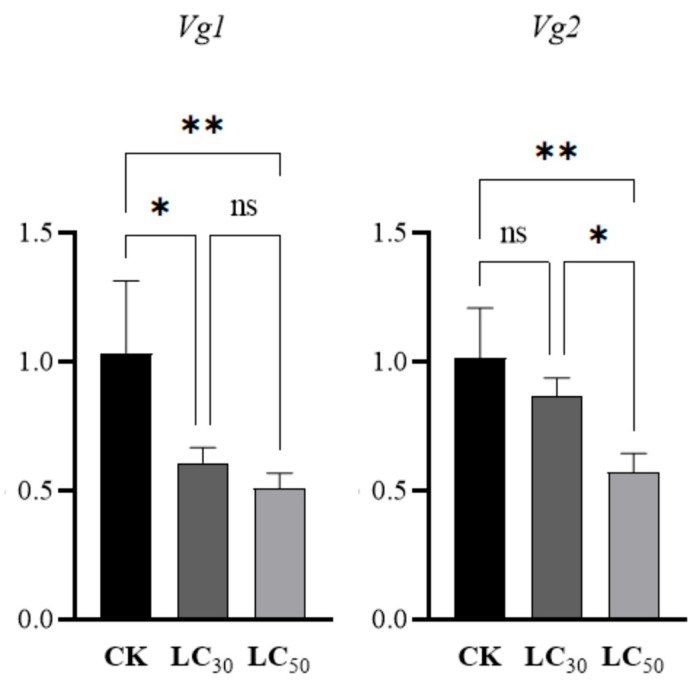
Relative expression levels of genes *NwVg1* and *NwVg2*. Note: ** indicates *p* < 0.01, * indicates *p* < 0.05 and ns indicates no significant difference.

In contrast, the relative expression level of the *NwVg2* gene decreased by 14.84% under the LC_30_ treatment, which was not significantly different (*p* > 0.05) from the control, but was significantly higher (*p* < 0.05) than that under the LC_50_ treatment. Furthermore, the LC_50_ treatment resulted in a 43.72% reduction in *NwVg2* expression, which was significantly lower (*p* < 0.01) than the control level (Figure 3).

### 3.6. RNA Interference (RNAi) of NwVg1 and NwVg2 Genes in N. womersleyi

#### 3.6.1. Validation of Gene RNAi Efficiency

The success of the RNAi procedure was confirmed through molecular validation. First, the synthesized dsRNA fragments were verified to be of the expected size (Figure S3). Subsequently, RT-qPCR analysis demonstrated that feeding of gene-specific dsRNA significantly suppressed the expression of both *NwVg1* and *NwVg2* compared to the dsGFP control (*p* < 0.001; Figure S4), achieving knockdown efficiencies of 81.68% and 80.14%, respectively. This confirmed the efficacy of the RNAi treatment prior to phenotypic analysis.

#### 3.6.2. Development and Reproduction of Female Adults Post-RNAi

The results of RNA interference are presented in Table 6. Compared with the control, dsRNA targeting *NwVg1* and *NwVg2* differentially affected various life parameters of F_0_ female adults. It should be noted that the parameter referred to here as “pre-oviposition period” measures the interval from the completion of the 24-h dsRNA feeding to the first oviposition, reflecting the time required for RNAi uptake and subsequent physiological impact.

One-way ANOVA revealed significant overall differences among treatments in female longevity (*F =* 68.34, *p* < 0.001), the post-feeding interval to first oviposition (*F* = 24.50, *p* < 0.001), oviposition period (*F* = 15.66, *p* < 0.001), and fecundity (*F* = 21.52, *p* < 0.001). Following *dsNwVg1* treatment, female longevity and fecundity were significantly reduced by 6.95% and 14.67%, respectively (*p* < 0.001), while the post-feeding interval to first oviposition was significantly prolonged by 50.00% (*p* < 0.001). The oviposition period did not differ significantly from the control.

After *dsNwVg2* treatment, female longevity, fecundity, and oviposition period were significantly reduced by 12.98%, 18.20%, and 8.77%, respectively (all *p* < 0.001), and the post-feeding interval was significantly extended by 47.00% (*p* < 0.001).

## 4. Discussion

Our integrated study reveals that sublethal pyridaben exposure induces a complex suite of transgenerational effects in *N. womersleyi*. A pivotal finding is the apparent paradox between clear stress signals—prolonged development, reduced immature survival, and a suppressed population growth rate (*r*)—and the stability of the net reproductive rate (*R*_0_) in the F_1_ generation. This juxtaposition forms the crux of our discussion, allowing us to address the ecological implications and, critically, to justify the mechanistic focus on vitellogenin (Vg) in light of the population-level resilience observed.

Ecological Implications: Costs, Compensations, and Unanswered Questions.

The significant prolongation of immature development in the F_1_ generation presents a dual-faceted ecological scenario. While conventionally viewed as a direct fitness cost that delays population recovery [28], it simultaneously raises a compelling hypothesis, as noted: an extended juvenile stage could increase total prey consumption, potentially offsetting individual costs at the level of ecosystem service (i.e., pest suppression) [29]. This underscores a critical gap in many ecotoxicological assessments: the need to evaluate the net ecological function of beneficial arthropods, not merely their life-history parameters. The concurrent reduction in immature survival observed here suggests that any potential behavioral compensation may be physiologically constrained by underlying stress, aligning with findings in other arthropods where sublethal exposure compromises survival under competitive or resource-limited conditions [30].

At the population level, the significant decline in *r* serves as a robust, integrative indicator of long-term population suppression risk. In contrast, the stability of *R*_0_, alongside the extended lifespan of F_1_ females, strongly hints at short-term physiological compensation. This phenomenon aligns with the concepts of adaptive homeostasis and hormesis, where organisms reallocate resources to buffer critical functions like reproduction under mild stress [31,32]. This compensatory capacity, however, raises a pivotal question for applied ecology: is such resilience sustainable across multiple generations in complex field environments, or does it mask a cumulative “physiological debt”?

The Role of Vitellogenin: A Molecular Probe into Compensated Toxicity.

The stability of *R*_0_ amidst significant downregulation of *NwVg1* and *NwVg2* is not a contradiction but a key insight. It demonstrates that Vg serves primarily as a sensitive “mechanistic probe”, precisely pinpointing the disruption of vitellogenesis-a cornerstone of reproductive investment as a specific molecular initiation point of pyridaben’s toxicity [33]. Its value lies in elucidating the mechanism of action, rather than acting as a direct, linear predictor of short-term population collapse in a compensatory context.

The activation of physiological compensation, inferred from stable *R*_0_, is decisively supported by our functional experiment. Targeted RNAi-mediated knockdown of *NwVg1*/*NwVg2* (acutely mimicking the chemical-induced molecular lesion) successfully recapitulated the core phenotype of reduced fecundity. This causal link confirms that Vg deficiency itself constitutes a genuine and substantial reproductive risk. The phenotypic differences between acute gene knockdown (e.g., delayed oviposition in adults) and chronic, multi-generational chemical exposure (prolonged development in immatures) are consistent with the distinct temporal and life-stage contexts of the perturbations, both ultimately stemming from interference with growth and reproductive pathways centered on Vg.

Evolutionary Conservation and Broader Context.

Our focus on *Vg* is further grounded in its evolutionarily conserved role in arthropod reproduction. Phylogenetic analysis confirmed that *NwVg1* and *NwVg2* are orthologs of functional *Vg* genes in related predatory mites (e.g., *N. barkeri* [34]) and other species, justifying their relevance as a study system. This evolutionary conservation carries significant ecotoxicological weight: the finding that pyridaben also suppresses *Vg* expression in the pest mite *Tetranychus cinnabarinus* [19] suggests that disrupting this pathway may represent a conserved mechanism of trans-species reproductive toxicity. This cross-species vulnerability highlights Vg as a high-value biomarker for a broadly relevant toxic pathway. Furthermore, the interspecific differences in sublethal response profiles—for instance, the prominent effect on adult longevity in *N. bicaudus* [35] versus developmental delay in *N. womersleyi* (this study)—underscore the necessity for species-specific ecological risk assessments.

## 5. Conclusions

In summary, this multi-level analysis reveals that sublethal pesticide exposure can simultaneously induce clear molecular and individual-level toxicity while being buffered at a key population-level reproductive endpoint in the short term. This has dual implications: for ecological risk assessment, it strongly advocates for incorporating sensitive molecular biomarkers like Vg as early-warning signals of physiological harm, which may precede or occur independently of immediate demographic changes. For integrated pest management (IPM), it serves as a caution that even in the absence of population collapse, the physiological costs incurred by natural enemies can undermine their long-term resilience and pest control efficacy. Future research should prioritize (1) direct, multi-generational tests of compensation sustainability, (2) integrative measurements of predation efficiency under sublethal stress to evaluate net ecological impact, and (3) exploration of the upstream regulatory networks of *Vg* to better predict and mitigate the sublethal impacts of agrochemicals on beneficial arthropods.

## Figures and Tables

**Table 1:** Fecundity and life span of female adult on *N. womersleyi* F_0_ generation treated with sublethal concentrations of pyridaben.

Parameter	Control	Pyridaben	
		LC_30_	LC_50_
Female longevity (d)	22.45 ± 0.18 a	18.13 ± 0.17 b	16.08 ± 0.14 c
APOP (d)	1.27 ± 0.06 c	1.62 ± 0.06 b	1.80 ± 0.05 a
Oviposition period (d)	15.77 ± 0.17 a	12.68 ± 0.11 b	11.63 ± 0.21 c
Average oviposition per female	41.73 ± 0.60 a	34.90 ± 0.37 b	18.08 ± 0.47 c

Note: The values in the table are presented as MEAN ± SE. Different letters after the data in the same row indicate significant differences (*p* < 0.05).

**Table 2:** Developmental periods of *N. womersleyi* F_1_ generation treated with sublethal concentrations of pyridaben.

	Parameter	Control	Pyridaben	
			LC_30_	LC_50_
Female	Egg duration (d)	1.00 ± 0 b	1.634 ± 0.076 a	1.692 ± 0.074 a
	Larva duration (d)	1.091 ± 0.043 a	1.588 ± 0.077 a	1.178 ± 0.072 a
	Protonymph duration (d)	1.00 ± 0 b	1.049 ± 0.035 ab	1.128 ± 0.054 a
	Deutonymph duration (d)	1.00 ± 0 a	1.00 ± 0 a	1.00 ± 0 a
	Preadult duration (d)	4.091 ± 0.043 b	5.268 ± 0.143 a	5.538 ± 0.126 a
	Longevity (d)	22.795 ± 0.452 b	24.707 ± 0.211 a	23.077 ± 0.201 b
	Total life span (d)	26.886 ± 0.453 c	29.976 ± 0.184 a	28.615 ± 0.165 b
Male	Egg duration (d)	1.00 ± 0 b	1.667 ± 0.113 a	1.842 ± 0.086 a
	Larva duration (d)	1.143 ± 0.105 b	1.50 ± 0.120 a	1.580 ± 0.115 a
	Protonymph duration (d)	1.071 ± 0.071 c	1.667 ± 0.113 a	1.216 ± 0.095 b
	Deutonymph duration (d)	1.00 ± 0 a	1.111 ± 0.075 a	1.00 ± 0 a
	Preadult duration (d)	4.214 ± 0.113 b	5.945 ± 0.334 a	5.632 ± 0.223 a
	Longevity (d)	27.071 ± 0.382 a	25.278 ± 0.538 b	24.00 ± 0.262 c
	Total life span (d)	31.286 ± 0.367 a	31.222 ± 0.365 a	29.632 ± 0.243 b

Note: The values in the table are presented as MEAN ± SE. Different letters after the data in the same row indicate significant differences (*p* < 0.05).

**Table 3:** Fecundity of *N. womersleyi* F_1_ generation treated with sublethal concentrations of pyridaben.

Parameter	Control	Pyridaben	
		LC_30_	LC_50_
APOP (d)	1.250 ± 0.086 a	1.205 ± 0.065 a	1.195 ± 0.062 a
TPOP (d)	5.341 ± 0.091 b	6.463 ± 0.154 a	6.744 ± 0.145 a
Oviposition period (d)	19.00 ± 0.807 a	18.00 ± 0.674 a	17.00 ± 0.761 a
Average oviposition per female	43.272 ± 1.254 a	41.951 ± 0.833 a	39.795 ± 0.606 b

Note: The values in the table are presented as MEAN ± SE. Different letters after the data in the same row indicate significant differences (*p* < 0.05).

**Table 4:** Effects on population parameters of *N. womersleyi* F_1_ generation treated with sublethal concentrations of pyridaben.

Parameter	Control	Pyridaben	
		LC_30_	LC_50_
Intrinsic rate of increase rate (d^−1^)	0.298 ± 0.086 a	0.263 ± 0.088 b	0.249 ± 0.091 b
Finite rate of increase (d^−1^)	1.348 ± 0.012 a	1.301 ± 0.0114 b	1.282 ± 0.116 b
Net reproductive rate (offspring/individual)	31.734 ± 2.627 a	28.667 ± 2.582 a	25.867 ± 2.487 a
Mean generation time (d)	11.591 ± 0.102 b	12.754 ± 0.125 a	13.085 ± 0.141 a
Doubling time (d)	2.324 ± 0.070 b	2.635 ± 0.090 a	2.788 ± 0.104 a

Note: The values in the table are presented as MEAN ± SE. Different letters after the data in the same row indicate significant differences (*p* < 0.05).

**Table 5:** Full-length sequence analysis results of *NwVg1* and *NwVg2*.

Gene Name	Accession Number	Sequence Length	Amino Acid Length	Relative Molecular Mass	Theoretical PI
*NwVg1*	OR897817.1	5538 bp	1663	465.54 kDa	4.60
*NwVg2*	OR897819.1	4341 bp	1445	350.33 kDa	4.72

**Table 6:** Fecundity and life span of female on *N. womersleyi* F_0_ generation treated with RNAi.

Parameter	Control	RNAi	
		*dsNwVg1*	*dsNwVg2*
Female longevity (d)	22.03 ± 0.24 a	20.50 ± 0.23 b	19.17 ± 0.18 c
Interval from dsRNA feeding to first oviposition (d)	1.00 ± 0.00 b	1.50 ± 0.09 a	1.47 ± 0.09 a
Oviposition period (d)	15.97 ± 0.19 a	15.60 ± 0.20 a	14.57 ± 0.18 b
Average oviposition per female	39.73 ± 0.96 a	33.90 ± 0.77 b	32.50 ± 0.97 b

Note: The values in the table are presented as MEAN ± SE. Different letters after the data in the same row indicate significant differences (*p* < 0.05).

## Data Availability

Researchers interested in accessing the data should contact the lead author via email juanwei666@163.com.

## Supplementary Materials

The following supporting information can be downloaded at: https://www.mdpi.com/article/10.3390/insects17010116/s1, Table S1: Primers for PCR; Table S2: Primers for qPCR; Table S3: Primers for dsRNA; Table S4: Bioinformatics analysis websites of *NwVg*; Table S5: Gene sequences used in phylogenetic analysis for *NwVg1* and *NwVg2*; Figure S1: Effects of sublethal concentrations of pyridaben on the population parameters of *N. womersleyi* F1 generation; Figure S2: Amplification products of *NwVg1* and *NwVg2* gene in *N. womersleyi*; Figure S3: Amplification products of RNAi segments; Figure S4: Relative expression levels of genes *NwVg1* and *NwVg2* after RNAi.
