# Supplementary material for: Effects of Sublethal Concentrations of Pyridaben on Development, Reproduction, and Vg Gene Expression in Neoseiulus womersleyi
Source: Insects. 2026 Jan 20;17(1):116. doi: 10.3390/insects17010116 (PMC12841668; doi:10.3390/insects17010116)
Supplement: Supplementary file 1 [file insects-17-00116-s001.zip › insects-3942201-supplementary.pdf]

Effects of Sublethal Concentrations of Pyridaben on Development, Reproduction, and *Vg* Gene Expression in *Neoseiulus womersleyi*

Juan Wei †, Chengcheng Li †, Cancan Song, Xinyue Yang, Chunxian Jiang * and Qing Li *

College of Agronomy, Sichuan Agricultural University, Chengdu 611130, China

* Correspondence: chunxianjiang@126.com (C.J.); liq8633@163.com (Q.L.)

† These authors contributed equally to this work.

SUPPORTING INFORMATION

Table S1. Primers for PCR

Gene name	Sequence (5'-3')
NwVg1-F	ATGAGATTCTTCCTCCCTCTTGT
NwVg1-R	TTACTGGGCGCGAACGCACT
NwVg2-F	ATCTCGACTTCGTGGTGGAC
NwVg2-R	CGAACTCAAGGGACAGACCG

Table S2. Primers for qPCR

Gene name	Sequence (5'-3')
NwVg1-F	GCAGGCTATCCGCAGAGGTA
NwVg1-R	GGGATGCTGACGAGGGTGTA
NwVg2-F	ATTTCGGTTTCGAGCTGACG
NwVg2-R	AGGTACTCCGAGCAGCGTCA
ACTB-F	TACGACCAGAAGCGTACAGC
ACTB-R	CCAACCGTGAAAAGATGACC

Table S3. Primers for dsRNA

Primers name	Sequence (5'-3')
dsNwVg1-F	<u>TAATACGACTCACTATAGGG</u><br>TCTACAACGTTCGCATCGAG
dsNwVg1-R	<u>TAATACGACTCACTATAGGG</u><br>ACCGTTCTGGAAGATGATGG
dsNwVg2-F	<u>TAATACGACTCACTATAGGG</u><br>GCCGACCTTGGTCATGTACT
dsNwVg2-R	<u>TAATACGACTCACTATAGGG</u><br>TCCAAGTGGGGAACTCAGAC
dsGFP-F	<u>TAATACGACTCACTATAGGG</u><br>GCCCGAAGGTTATGTACAGG
dsGFP-R	<u>TAATACGACTCACTATAGGG</u><br>CTTTTCGTTGGGATCTTTCG

Table S4. Bioinformatics analysis websites of *NwVg*

Analysis contents	Websites address
sequence alignment	https://blast.ncbi.nlm.nih.gov/Blast.cgi?PROGRAM=blastn&PAGE_TYPE=BlastSearch&LINK_LOC=blasthome
Coding protein prediction	https://web.expasy.org/protparam/
Conservative domain prediction	http://www.ncbi.nlm.nih.gov/Structure/cdd/wrpsb.cgi

Table S5. Gene sequences used in phylogenetic analysis for *NwVg1 and NwVg2*

Species name	Accession number
Neoseiulus barkeri	KX620366.1
Neoseiulus barkeri	KX620367.1
Euseius nicholsi	MN555331.1
Euseius nicholsi	MK135169.1
Neoseiulus cucumeris	KC456602.1
Neoseiulus cucumeris	KC456603.1
Amblyseius eharai	MN555329.1
Amblyseius eharai	MH113158.1
Dermanyssus gallinae	OR601170.1
Panonychus citri	XM_053351153

Note: The GenBank accession number starting with the XM_ is based on the predicted sequence derived from the genome sequence.

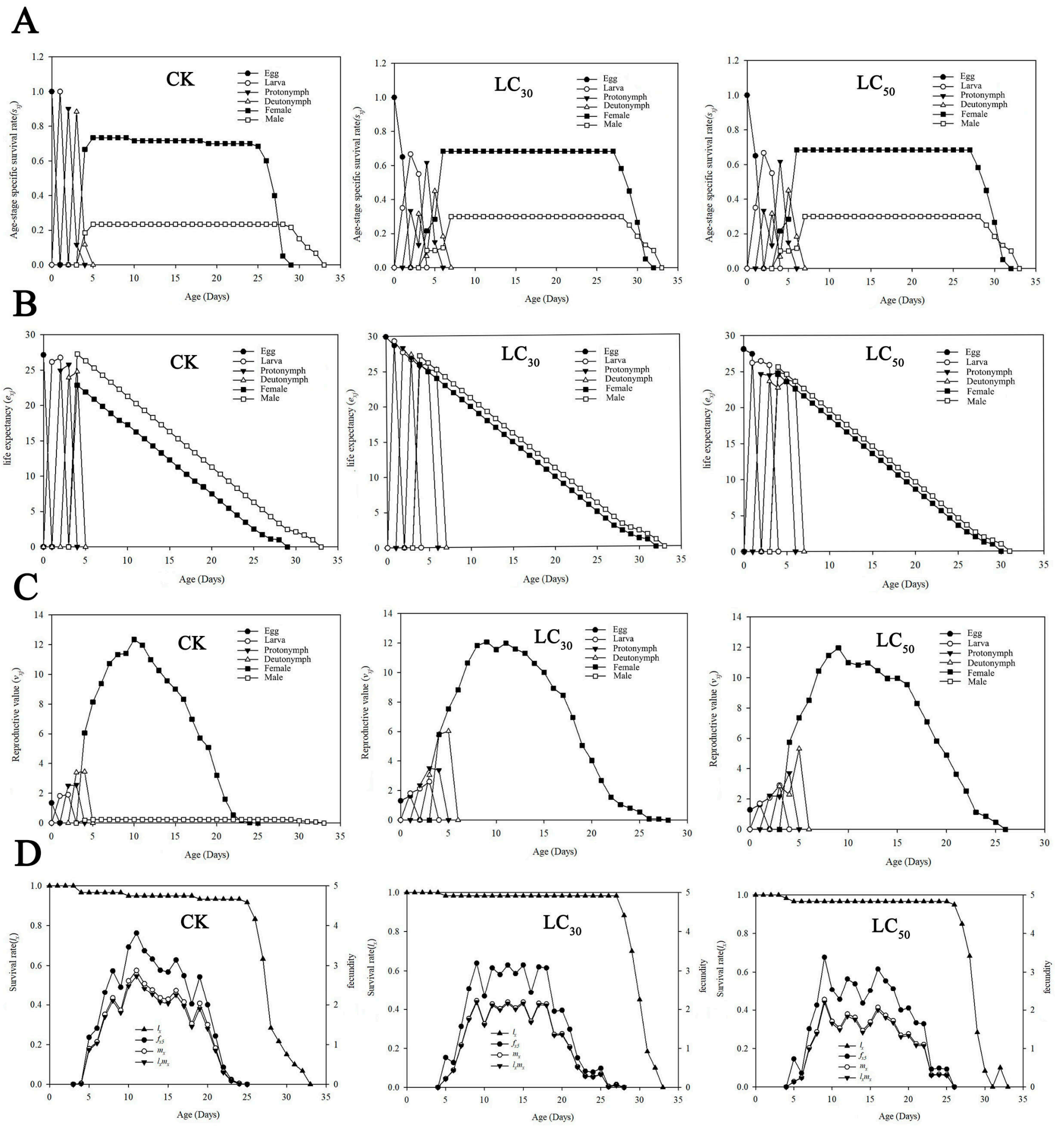

Figure S1. Effects of sublethal concentrations of pyridaben on the population parameters of *N. womersleyi* F_1 generation. **(A)**: Age-stage specific survival rate (S_{xj}) of *N. womersleyi* F_1 generation treated with sublethal concentrations of pyridaben. **(B)**: Age-stage life expectation (S_{xj}) of *N. womersleyi* F_1 generation treated with sublethal concentrations of pyridaben. **(C)**: Age-stage reproductive values (v_{xj}) of *N. womersleyi* F_1 generation treated with sublethal concentrations of pyridaben. **(D)**: Age-specific survival rate (l_x), female age-specific fecundity (f_{x5}), age-specific fecundity of total population (m_x), and age-specific maternity (l_xm_x) of *N. womersleyi* F_1 generation treated with sublethal concentrations of pyridaben.

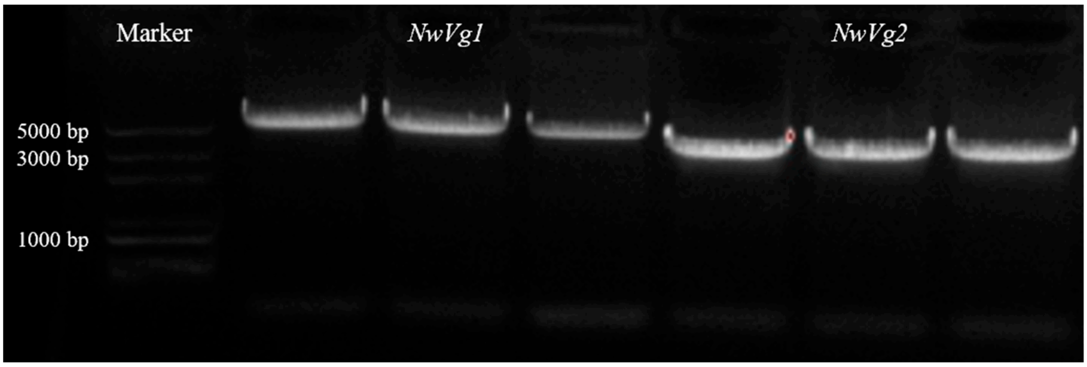

Figure S2. Amplification products of *NwVg1* and *NwVg2* gene in *N. womersleyi.*

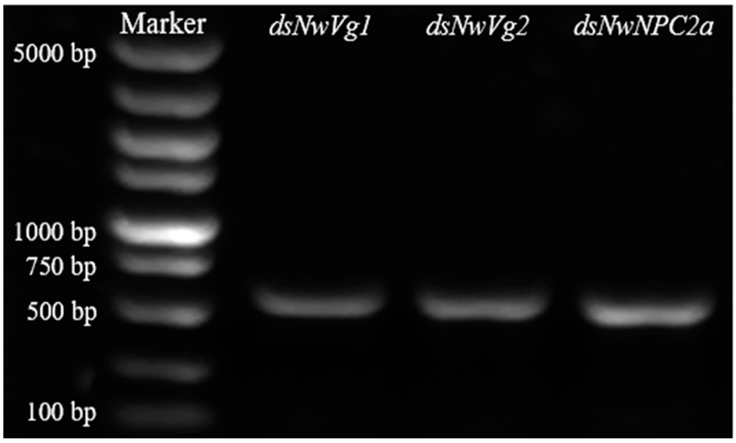

Figure S3. Amplification products of RNAi segments.

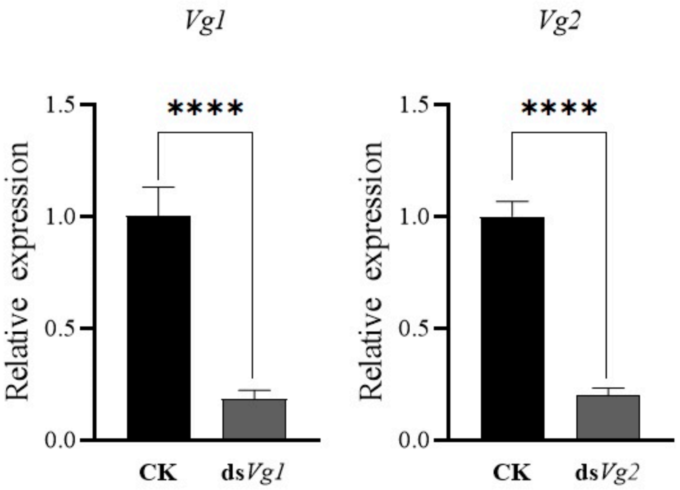

Figure S4. Relative expression levels of genes *NwVg1* and *NwVg2* after RNAi.

1
